# Oberlin transfer for C5-6 palsy after posterior cervical spine surgery

**DOI:** 10.3171/2022.10.FOCVID22100

**Published:** 2023-01-01

**Authors:** Stephen P. Miranda, Jessica Nguyen, Sanjana Salwi, Eric L. Zager, Zarina S. Ali

**Affiliations:** Department of Neurosurgery, Hospital of the University of Pennsylvania, Philadelphia, Pennsylvania

**Keywords:** Oberlin procedure, nerve transfer, C5–6 palsy, cervical spine surgery

## Abstract

Postoperative C5–6 palsies can occur in 5%–10% of cases after cervical spine surgery. In this video, the authors demonstrate operative techniques for nerve transfer to restore function for postoperative C5–6 palsy. The patient underwent C3–6 laminectomy and posterior fusion for cervical spondylotic myelopathy and developed weakness postoperatively in the C5–6 distribution bilaterally. He experienced spontaneous recovery to near full strength in the most affected muscle groups by 12 months except the left biceps (2/5), with at least antigravity shoulder abduction. He underwent left ulnar to musculocutaneous nerve fascicular transfer to improve elbow flexion and supination in the setting of good hand function.

The video can be found here: https://stream.cadmore.media/r10.3171/2022.10.FOCVID22100

## Transcript

In this video, we demonstrate the operative techniques for the Oberlin procedure to restore function for C5–6 palsy after posterior cervical spine surgery.

### 0:30 Background.

Postoperative C5–6 palsies can occur in 5%–10% of cases after cervical spine surgery.^1–3^ Nerve transfer has been shown to restore function for those with persistent weakness in retrospective case series. In one such series, 9 of 10 treated patients had recovery in biceps and deltoid function at last follow-up.^4^ Possible nerve transfers include spinal accessory to suprascapular nerve transfer, triceps branch of the radial nerve to axillary nerve transfer, as well as single or double fascicular transfer from the ulnar and/or median nerve to the biceps and/or brachialis motor branches of the musculocutaneous nerve, which is known as the Oberlin procedure.^5,6^

### 1:09 Patient Presentation.

The patient is a 55-year-old right-handed male who initially presented with a 1-year history of gait instability, subjective right lower-extremity weakness, dysesthesias in the bilateral feet, and intermittent radiculopathy in the bilateral upper extremities, most notably in the C6 distribution. On examination, he had subtle hand intrinsic weakness bilaterally, hyperreflexia in all four extremities, and abnormal tandem gait, concerning for cervical spondylotic myelopathy. Imaging studies demonstrated multilevel degenerative changes resulting in cervical stenosis between C3–6 with evidence of cord signal change. Given evidence of partial ossification of the posterior longitudinal ligament, he was counseled against multilevel anterior cervical discectomy and fusion and ultimately underwent C3–6 laminectomy and posterior fusion. Postoperative imaging studies are shown here, demonstrating successful decompression and stabilization.

### 2:02 Postoperative Bilateral C5–6 Palsy.

Postoperatively, the patient reported that his radiculopathy and gait had significantly improved; however, he developed bilateral upper-extremity weakness in the C5–6 distribution over a period of days: his right deltoid was 2/5, his left deltoid was 1/5, and bilateral biceps were 2/5, with weakness also noted in the left supra- and infraspinatus.

### 2:25 EMG Findings (10 Months Postoperatively).

The patient was followed closely with both serial neurological examinations and electrodiagnostic studies averaging every 3 months, and he was noted to have spontaneous recovery of function to near full strength in all affected muscle groups of the right upper extremity by 10 months. An electromyography study demonstrated motor unit potentials in the left biceps, but he still could not achieve antigravity function.

### 2:48 Examination (12 Months Postoperatively).

At 12 months, in the left upper extremity, his supra- and infraspinatus were 4/5, his deltoid was 3/5, and he was able to achieve adequate shoulder abduction. He still exhibited weakness in the biceps (2/5) but was able to flex at the elbow in a neutral position predominantly through use of his brachioradialis.

### 3:16 Surgical Intervention.

The patient was offered an Oberlin procedure on the left side to improve elbow flexion and supination in the setting of good hand function, via an ulnar nerve to musculocutaneous nerve transfer. While nerve transfers for improvement of shoulder abduction were considered, including radial to axillary nerve transfer and spinal accessory to suprascapular nerve transfer, the authors do not believe that patients with at least antigravity function are appropriate candidates for these additional transfers. However, these options are often used in conjunction with the Oberlin transfer when patients do have severe shoulder abduction and/or external rotation weakness following posterior cervical decompression surgery.

The patient was taken to the operating room and the left medial arm was marked. After general endotracheal anesthesia was induced, the patient was positioned supine on the operating table with the left medial arm abducted and externally rotated onto a hand table. A linear incision was marked overlying the neurovascular bundle between the atrophied biceps and the triceps muscle. The skin was infiltrated with local anesthetic and an incision was opened with a No. 15 blade. Dissection then proceeded through the subcutaneous tissues and fascia to the neurovascular bundle. The medial antebrachial cutaneous nerve and medial cutaneous nerve of the forearm were identified and protected. Exposure continued medially where the ulnar nerve was identified and neurolysed with good response to stimulation. Just lateral to the brachial artery, the median nerve was then identified, also with good response to stimulation. Next, the interval between the atrophied biceps and brachialis muscles was explored to identify the musculocutaneous nerve. These nerves were dissected and encircled with vessel loops. The proximal branch of the musculocutaneous nerve was then isolated and neurolysed and encircled with a vessel loop. There was minimal electrical response to stimulation of this branch, as expected from his preoperative examination. The operating microscope was brought into the field and microsurgical technique was used to open the epineurium of the ulnar nerve. An internal neurolysis was performed of the ulnar nerve with stimulation of fascicles. We identified one fascicle that provided response mainly in the flexor carpi ulnaris, more so than hand function, at very low current. This fascicle was gently dissected from the remaining ulnar nerve fascicles using microsurgical technique and was isolated with a vessel loop so that it could be dissected distally for a significant distance. The biceps branch of the musculocutaneous nerve was then sectioned proximally and brought downward and medially toward the ulnar nerve. The selected ulnar nerve fascicle was transected distally and was brought toward the biceps branch. The fascicular nerve repair was then performed using a single interrupted suture of 8-0 nylon with no tension at the suture line. There was a good size match and good apposition of the nerve ends. The repair site was covered with fibrin glue. The wound was then irrigated with saline and meticulous hemostasis was obtained. The incision was then closed in anatomical layers using interrupted 3-0 absorbable sutures and a running 3-0 subcuticular suture for the skin closure. A dry sterile dressing was placed, a compressive wrap was applied, and a sling was placed on the arm. The patient was extubated and taken to the recovery room postoperatively in satisfactory condition.

### 8:21 Postoperative Care.

The patient kept his arm in a sling until his postoperative visit at 3 weeks, to avoid disruption of the coaptation. His incision had healed well. He reported minimal pain at the incision but had mild anterior forearm numbness. He has follow-up scheduled at 6 months postoperatively as well as at 9 months for repeat electromyography and nerve conduction studies to monitor the outcome of this nerve transfer.

## Disclosures

The authors report no conflict of interest concerning the materials or methods used in this study or the findings specified in this publication.

## Author Contributions

Primary surgeon: Ali. Assistant surgeon: Salwi, Zager. Editing and drafting the video and abstract: Miranda, Nguyen, Ali. Critically revising the work: Miranda, Zager, Ali. Reviewed submitted version of the work: Miranda, Salwi, Zager, Ali. Approved the final version of the work on behalf of all authors: Miranda. Supervision: Zager. Captured all video recording of surgery: Nguyen.

## References

[1] Bydon M Macki M Kaloostian P Incidence and prognostic factors of C5 palsy: a clinical study of 1001 cases and review of the literature Neurosurgery 2014 74 6 595 605 24561867 10.1227/NEU.0000000000000322

[2] Nassr A Eck JC Ponnappan RK Zanoun RR Donaldson WF III Kang JD The incidence of C5 palsy after multilevel cervical decompression procedures: a review of 750 consecutive cases Spine (Phila Pa 1976) 2012 37 3 174 178 22293780 10.1097/BRS.0b013e318219cfe9

[3] Shou F Li Z Wang H Yan C Liu Q Xiao C Prevalence of C5 nerve root palsy after cervical decompressive surgery: a meta-analysis Eur Spine J 2015 24 12 2724 2734 26281981 10.1007/s00586-015-4186-5

[4] Lubelski D Pennington Z Kopparapu S Nerve transfers after cervical spine surgery: multi-institutional case series and review of the literature World Neurosurg 2021 156 e222 e228 34536618 10.1016/j.wneu.2021.09.039

[5] Oberlin C Béal D Leechavengvongs S Salon A Dauge MC Sarcy JJ Nerve transfer to biceps muscle using a part of ulnar nerve for C5-C6 avulsion of the brachial plexus: anatomical study and report of four cases J Hand Surg Am 1994 19 2 232 237 8201186 10.1016/0363-5023(94)90011-6

[6] Ray WZ Pet MA Yee A Mackinnon SE Double fascicular nerve transfer to the biceps and brachialis muscles after brachial plexus injury: clinical outcomes in a series of 29 cases J Neurosurg 2011 114 6 1520 1528 21351836 10.3171/2011.1.JNS10810

